# Subjective burdens among informal caregivers of critically ill patients: a cross-sectional study in rural Shandong, China

**DOI:** 10.1186/s12904-021-00858-4

**Published:** 2021-10-22

**Authors:** Wenhao Fu, Jiajia Li, Feng Fang, Dan Zhao, Wenting Hao, Shixue Li

**Affiliations:** 1 Centre for Health Management and Policy Research, School of Public Health, Cheeloo College of Medicine, Shandong University, Jinan, 250012 China; 2 NHC Key Lab of Health Economics and Policy Research (Shandong University), Jinan, 250012 China

**Keywords:** Informal caregiver, Subjective burden, Patients with critical illness, China

## Abstract

**Background:**

Informal caregivers are the main source of care for the critically ill, especially after discharge or during the terminal stages at home. However, the concern for informal caregivers is often overshadowed by critically ill patients. The purpose of this study is to determine the influencing factors of the subjective burden of informal caregivers and to seek solutions accordingly.

**Methods:**

Between July and August 2019, a cross-sectional study was conducted in Shandong, China, focusing on family caregivers and critically ill patients. Subjective caregiver burden was measured by the Chinese version of Zarit Burden Interview (ZBI). The stress process model was used to identify conditions relevant to the caregiving burden and to assess their impact on family caregivers.

**Results:**

554 samples were selected for analysis. The average scores of Zarit Caregiver Burden Interview (ZBI) scores in this study was 30.37±19.04 (n=554). ZBI scores of older, less educated, and spouse caregivers were significantly lower (4.12; 95%CI, 0.42 to 7.81; P =0.029). Objective and subjective burdens increased proportionally. Secondary role stress factors included the higher out-of-pocket (OOP) costs of critical diseases and lower household income, both of which increased caregivers’ subjective burdens (1.28; 95%CI, -0.06 to 2.63; p=0.062). Formal medical aid systems played a positive role in reducing subjective caregiving burdens (-7.31; 95%CI, -13.23 to -1.40; p=0.016).

**Conclusions:**

Health policies should address both the direct medical burdens and the intangible psychological burdens of critical diseases.

## Background

As patient loads grow, informal care is an increasingly important part of health care systems. An informal caregiver provides unpaid support to a family member or friend [1, 2]. Informal caregivers provide approximately 90% of long-term care for adults living at home in the United States [3], and an estimated 80% of care for non-self-sufficient patients in Europe [4]. Informal caregivers generally shoulder a heavier stress burden than formal nurses, due to longer hours, emotional exhaustion, and personal financial burden shared with their patients [5–8]. Such stress is known as “secondary role strains” in Pearlin’s stress process model [9]. Informal caregivers suffer more health problems and depressive symptoms, as well lower self-reported family health than formal nurses [10–14]. This suffering is particularly acute among caregivers for patients suffering from critical illnesses, which has been described as ‘we-disease’ [13].

Critical illnesses are long-lasting, difficult-to-cure diseases with high financial costs that alter the lives of patients and their families over a long period of time [15–17]. In addition to causing physical injury and psychological stress to patients themselves, critical illnesses are also one of the main causes of patients’ families falling into poverty [14, 18].

Family caregivers are the main source of care for the critically ill, especially after discharge from a hospital or during terminal stages at home. Caregivers of critically ill patients shoulder multidimensional challenges, including monitoring diseases, providing emotional support, and sharing financial burdens [19]. Such prolonged challenges may result in depression, stress, diminished physical health, and even an increased risk of heart disease [20–22]. The caregiving capacity and efficiency of informal caregivers may thus be impaired, interfering with the recovery of critically ill patients and creating a vicious cycle.

Caregiver burden is defined as a negative reaction to the impact of providing care on the caregiver’s social, occupational, and personal roles [23]. In 1966, Hoenig and Hamilton extended this concept into subjective burden and objective burden [24]. The objective burden refers to the observable, tangible cost caused by the care- recipients’ illness to the caregiver [25], while the subjective burden refers to the personal perception and personal evaluation of the extent of caregiving burden [24, 25]. The subjective burden arising from caring for a frail or disabled relative can lead to emotional, mental, and physical health problems for caregivers [26, 27], however, may be overlooked by researchers because they are not directly visible [24]. Therefore, we need to pay more attention to the subjective burden.

Unfortunately, the subjective burden of caregivers can be unappreciated [28]. Even in countries like the United States, caregivers often lack direct support either as caregivers or as individuals [29]. Caregiver burden has been an issue of concern, with female gender, lower educational attainment, depression symptoms, social isolation, financial stress, and poor health status being risk factors for caregiver burden [30]. Among informal caregivers, Sspousal caregivers may shoulder morewere more likely to experience caregiver burden than other family members [31]. For caregivers of cancer patients, caregiver burden was heavier for those who were younger, male, single, and with primary school education or below [32]. However, Intas et. al., found that caregiving burden was not associated with caregiver’s age or years of care for family caregivers of hemodialysis patients with chronic kidney disease-end stage. Han et al. 's study on the care burden of family caregivers of stroke survivors found that caregiver age and depressed mood, as well as care duration, were determinants of caregiver burden [33]. From the perspective of patient characteristics, existing studies found that their education level, functional status were not significantly associated with caregiver burden [12, 34, 35]. However, studies linking caregiver factors to patient factors that directly affect the intensity of care were fall from enough.

Moreover, although the government has carried out the Critical Illness Insurance to relieve the economic burden of the families with critically ill patients, their caregiving burden has not been paid enough attention by policy and research. We have limited knowledge of how to reduce the burden of the family caregivers for patients suffering from critical illness, particularly in the context of rural China. Professional nursing institutions and nursing staff are in short supply in China, especially in rural areas. Data from China Health Statistics Yearbook showed that there were only 2 nurses per 1000 in rural areas in 2019 [36]. Therefore, it is necessary to conduct a study in China that incorporates both information on patients with critical illness and their informal caregivers, as well as their family information, to comprehensively explore the factors associated with informal caregiver burden in rural patients with critical illness.

The purpose of this study was to answer what factors might be associated with caregiving burdens of the critically ill patients and to seek solutions accordingly. The underlying study applies and interrogates the stress process model, care need factors, objective burdens, secondary role strain factors, and caregiver backgrounds. Understanding the potential effects of protective and risk factors will help formulate targeted interventions to improve outcomes for family caregivers of critically ill patients.

## Methods

### Study design and setting

Between July and August 2019, a cross-sectional study was conducted in Shandong, China, a central province with a population of more than 100 million. Yantai, Weifang, and Heze were selected as representative regions from the eastern, central, and western parts of Shandong province, based on their socio-economic development, health resource distribution, population structures, and geography.

### Study population

Informal caregivers were defined as those who (i) look after and assume primary responsibility for the care of patients; (ii) aged 18 years and above; (iii) not be paid ; (iiii)care critically ill patients and (iiiii) able to understand the contents of the interview and can communicate normally with the interviewer during the interview. Critically ill patients were defined as meeting at least one of the following criteria: 1) families with patients whose OOP (out-of-pocket) expenses exceed the local Critical Illness Insurance (CII) threshold; 2) Even if the single-phase OOP did not exceed the threshold, families with patients who suffered from diseases that are long-lasting and seriously impacts the patient's ability to obtain income or engage in daily living, such as hematopoietic stem cell transplantation and end-stage renal disease, sequelae of stroke and severe mental illness.

### Sampling method

Patients with critical illness were selected in Shandong Province by stratified cluster sampling. Using the family as the unit, researchers approached informal caregivers who met the criteria and the corresponding patients with critical illness, respectively. The researchers provided them with an explanation about the study. Those willing to participate in the interview were asked to sign an informed consent form.

### Study procedure and process

**Fig. 1:**
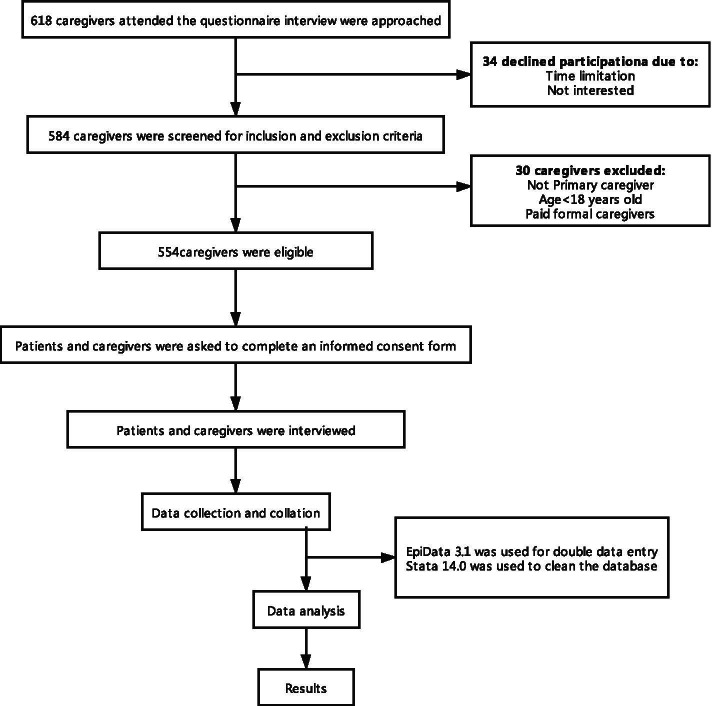
Flow chart of the conduct of the study

Caregivers answered questionnaires separately from corresponding patients with critical illness. Considering the generally low educational attainment of the rural residents, we took a face-to-face survey and interviewed, explained, and filled out questionnaires by the investigators. The investigators were divided into four groups, each of which checked the others for logical contradictions, missing data, and other problems at the end of each day. Questionnaire information was taken in the form of an interview filled out by a trained interviewer, avoiding illiteration of the respondent, and the occurrence of other errors in filling out the questionnaire. The interview took approximately 35-40 minutes to complete the questionnaire. Fig.1 illustrates the conduct of the study. EpiData 3.1 was used for double data entry to ensure data accuracy. Stata 14.0 was used to clean the database.

### Study tools

**Fig. 2:**
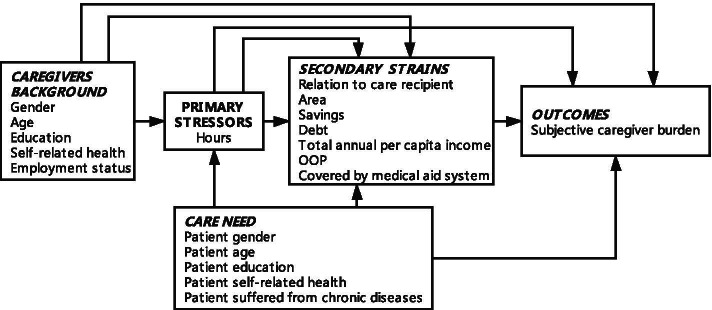
Stress process model for overview of the study aims

The caregiver stress process model proposed by Pearlin et al., which identifies perceived caregiver stress associated with caring for older adults, were served as a precise theoretical framework for understanding caregiver burden [9, 37, 38]. . Combined Tough's dyadic perspective with Pearlin's stress process model, five aspects were included in our study, including caregivers’ background, care need, primary stressors, secondary strains, outcome [26]. According to Pearlin’s and Judge’s study family caregiver burdens derive from the primary stressor of providing sustainable care for patients, often involving patients’ socioeconomic characteristics, resources, and self-rated health [26]. We use the subjective burden of caregiving as our dependent variable to represent the outcome. With reference to the study by Souza [20], Groenou [39] and Chou [40], Caregiving hours (objective burden) and patient care need (patient gender, patient age, patient education, patient self-related health, patient suffered from chronic diseases) were used as metrics for the primary stressor. The secondary role strain was assessed through a comprehensive set of indicators, including relationship with the care recipient, caregiving time, geographical location, savings, debt, total annual per capita income, OOP expenses, and medical system coverage (Fig. 2) [41]. Additionally, the caregivers’ demographics (such as age, gender, and education) and their self-assessed health and employment statuses were incorporated into the model according to Tough’s [26], Zarit [42], Oldenkamp [43] and Judge [38].

According to tough's research [26, 44], subjective burden refers to the emotional or psychological impact on caregiving tasks on caregivers, and objective burden refers to the amounts of activities the caregiver provides, as well as the time burden of providing support for these tasks. In this study, we defined the subjective burden as caregiver personal perceived burden and the objective burden as daily care time.

We employed the Chinese version of Zarit Burden Interview (ZBI), a widely used scale with proven statistical power [45–47], to measure the subjective burden of caregivers. The scale includes 22 questions rated on a 5-point scale [42, 48]. Caregivers assigned a numerical value between 0 to 4 for each aspect of their burdens, with higher numbers corresponding to higher perceived burdens. The ZBI summary score combines the responses to generate a numerical overall subjective burden ranging from 0 to 88. According to Lu et. al (2009) [48], the Chinese version of ZBI had high internal consistency(Cronbach’s α=0.875, KMO=0.867), and goodness of fit in confirmatory factor analysis (RMSEA = 0.077; CFI = 0.841; NFI = 0.802; GFI = 0.886), which proved the good reliability and validity of the Scale [44]. In this study, Cronbach’s alpha of the scale was 0.943 (p<0.000) and KMO of the scale was 0.935 (p<0.000).

### Sample size calculation

The sample size calculation was performed by the following formula:$N=\frac{u_{\alpha\left/{2} \right.}^2\pi \left(1-\pi \right)}{\delta^2}$ (*π*: overall rate) [49]. Because the rate of patients with critical illness and their caregivers is unknown in the full population. We assume that the π=0.5 [49, 50]. In this study, $\pi =0.5,{u}_{\alpha\left/{2}\right.}=1.96,\delta =0.1\pi =0.05$. The calculated sample size was 385. Considering 20% non-responders [51], we found a minimum sample size of 462. A stratified cluster sampling method was used. Samples with missing exclusion information as well as those that did not meet the inclusion criteria were excluded. We finally obtain 554 valid questionnaire responses from 77 villages.

### Ethical considerations

Before the investigation, signed informed consent was obtained from each prospective participant. The Academic Research Ethics Committee of Shandong University validated this form of consent and approved the research proposal.

### Statistical analysis

Data was analyzed using Stata 14.0. Normality of the variables was verified using the Shapiro-Francia W' test prior to analysis. Categorical variables were presented with absolute and relative frequencies, and continuous variables were presented with median and IQRs. Wilcoxon rank sum test for binary variables, Kruskal Wallis rank sum test for polytomous variables, and Spearman rank correlation test for continuous variables were used to identify factors that were significantly associated with caregiver burden. OLS regression was employed to explore these associations after controlling the confounding factors. Multicollinearity was tested using the variance inflation factor (VIF). The VIF of each variable was less than 10, which proved no multicollinearity existed.

## Results

### Descriptive results

The average ZBI scores in this study was 30.37±19.04 (n=554). Sightly more than half of informal caregivers were female (50.90%) and unemployed (53.61%), as shown in Table 1. 53.79% of family caregivers were 65 years old or younger, 59.21% had at least a junior high school education, and 79.24% had good self-rated health. A supermajority of informal caregivers were spouses (79.78%) whose caregiving time generally averaged under12 h/d (63.36%). Most surveyed families had no savings (63.18%) and no debt (58.3%). Even though the sampled patients were all critically ill, 93.68% were not covered by any medical aid system.

**Table 1:** Informal caregivers, Patients and Secondary role strains Characteristics (N = 554)

Variable	Burden interview		
	N (%)	Test Statistic	P
**Caregivers**			
Gender			
Male	272(49.10)	-0.11	0.916
Female	282(50.90)		
Age			
65 and below	298(53.79)	2.41	0.016
66 and above	256(46.21)		
Education			
Primary schools and below	226(40.79)	-0.82	0.415
Secondary and above	328(59.21)		
Self-rated health			
Good	439(79.24)	1.98	0.048
Poor	115(20.76)		
Employment status			
Employment	257(46.39)	0.68	0.499
Unemployment	291(53.61)		
Caregiver			
Spouse	442(79.78)	-2.40	0.016
Other relatives	112(20.22)		
**Objective burden**			
Caregiving hours per day			
≤4	170(30.69)	19.58	0.000
5-8	81(14.62)		
9-12	100(18.05)		
>13	203(36.64)		
**Patients with critical illness**			
Gender			
Male	275(49.64)	0.53	0.605
Female	279(50.36)		
Age			
65 and below	280(50.54)	0.125	0.900
66 and above	274(49.46)		
Education			
Illiterate	113(20.40)	-1.82	0.069
Educated	441(79.60)		
Self-rated health			
Good	251(45.31)	7.72	0.000
Poor	303(54.69)		
Whether suffered from chronic diseases			
Yes	194(35.02)	1.92	0.138
No	360(64.98)		
**Secondary stressor**			
Savings			
Yes	204(36.82)	1.53	0.055
No	350(63.18)		
Debt			
Yes	231(41.70)	5.884	0.000
No	323(58.30)		
Total annual per capita income^a^			
Q1	139(25.09)	36.14	0.000
Q2	137(24.73)		
Q3	139(25.09)		
Q4	139(25.09)		
Whether covered by medical aid system			
No	519(93.68)	0.72	0.473
Yes	35(6.32)		
Log OOP of critical illness (Median, IQR)	10.30(9.66,10.82)	Spearman's rho= 0.1183	0.005
Area			
Yantai	187(33.75)	11.69	0.003
Weifang	225(40.61)		
Heze	142(25.63)		

Note: ^a^ Quartile 1 (Q1) is the poorest and Quartile 4 (Q4) is the richest

### Univariate analysis

Summary statistics illustrated that caregiver age, patient relationship, hours spent caregiving, family debt, total annual per capita income, OOP costs, and their patient’s self-rated health were all significantly associated with the ZBI score of family caregivers (Table1).

### Multivariate regression analyses

OLS regression analysis model results are illustrated in Table 2. Younger caregivers (aged 65 and below) and more educated caregivers (with at least secondary schooling) faced greater subjective burdens than their counterparts, with significance of -6.05; 95%CI,10.68 to -1.42; P=0.011 and 3.61; 95%CI, 0.49 to 6.73; p=0.023, respectively. ZBI scores of spouse caregivers were significantly lower than those of other family caregivers (4.12; 95%CI, 0.42 to 7.81; P =0.029).

**Table 2:** OLS regression analysis of informal caregivers’ burden related factors

Factors	β	SE	t	P-Values	95% CI for β
Gender of caregivers: Female (ref: Male)	-0.54	2.07	-0.26	0.795	(-4.60, 3.52)
Age of caregivers: 66 and above (ref: 65 and below)	-6.05	2.36	-2.57	0.011	(-10.68, -1.42)
Education of caregivers: Secondary and above (ref: Primary schools and below)	3.61	1.59	2.27	0.023	(0.49, 6.73)
Self-rated health of caregivers: Poor (ref: Good)	-1.33	1.87	-0.71	0.477	(-5.01, 2.34)
Employment status: Unemployment (ref: Employment)	-0.66	1.55	-0.42	0.672	(-3.71, 2.39)
Caregiver: other relatives (ref: Spouse)	4.12	1.88	2.19	0.029	(0.42, 7.81)
Caregiving Time (ref: ≤4 )					
5-8	-0.22	2.30	-0.10	0.923	(-4.75, 4.30)
9-12	4.41	2.15	2.05	0.041	(0.18, 8.63)
>13	5.02	1.83	2.75	0.006	(1.44, 8.61)
Gender of patients: Female (ref: Male)	-2.56	2.03	-1.26	0.207	(-6.55, 1.42)
Age of patients: 66 and above (ref: 65 and below)	5.52	2.34	2.36	0.018	(0.93, 10.11)
Education of patients: Educated (ref: Illiterate)	2.99	1.98	1.51	0.131	(-0.90, 6.88)
Self-rated health of patient: Poor (ref: Good)	10.61	1.58	6.72	0.000	(7.51, 13.71)
Patients suffered from chronic diseases: No (ref: Yes)	-5.31	2.49	-2.13	0.033	(-10.19, -0.42)
Savings: No (ref: Yes)	-1.52	1.58	-0.96	0.336	(-4.61, 1.58)
Debt: No (ref: Yes)	-6.88	1.63	-4.22	0.000	(-10.08, -3.68)
Total annual per capita income (ref: Q1)^a^					
Q2	-5.10	2.08	-2.45	0.015	(-9.20, -1.01)
Q3	-7.20	2.07	-3.47	0.001	(-11.26, -3.13)
Q4	-11.13	2.16	-5.16	0.000	(-15.37, -6.90)
Log OOP	1.28	0.69	1.87	0.062	(-0.06, 2.63)
Covered by medical aid system: Yes (ref: No)	-7.31	3.01	-2.43	0.016	(-13.23, -1.40)
Area (ref: Yantai)					
Weifang	-3.70	1.76	-2.10	0.036	(-7.15, -0.24)
Heze	-0.22	2.09	-0.10	0.918	(-4.31, 3.88)

Note: ^a^ Quartile 1 (Q1) is the poorest and Quartile 4 (Q4) is the richest

In addition, subjective burdens were proportional to objective burdens. For example, the ZBI of caregivers who spent more than 13 hours per day caring for patients was significantly higher than that of other caregivers (5.03; 95%CI, 1.44 to 8.61; P=0.006). Higher OOP costs of critical diseases, the heavier subjective burden of the caregivers (1.28; 95%CI, -0.06 to 2.63; p=0.062). Meanwhile, as family per capita income improved, the subjective burden of informal caregivers decreased. Formal medical aid systems also played a positive role in reducing subjective caregiving burdens (-7.31; 95%CI, -13.23 to -1.40; p=0.016). Finally, the subjective caregiving burdens increased when care recipients were older (aged 66 years and above (P=0.884), had poorer self-reported health (P=0.000), or had other chronic diseases in addition to their critical illnesses (P=0.138).

## Discussion

Critical illnesses have influence both patients and their families. Our study focused on the informal caregivers of patients with critical illnesses and found that more than a quarter of family caregivers faced a moderate to severe burden with a ZBI greater than 40. The average ZBI score in this study was 30.37±19.04. Subjective caregiver burden in our study were heavier than those reported other Chinese studies on oncology, rheumatoid arthritis, and schizophrenia, in which caregivers generally had a mild or moderate burden [52–54]. One possible explanation is that the population involved in this study was caregivers of critically ill patients in rural areas, who have relatively lower incomes and fewer assets and therefore shoulder heavier financial burdens. Additionally, the health status of cancer patients may change suddenly and unpredictably, and this uncertainty increases subjective care burden for family caregivers [55]. Other studies of informal caregivers for advanced cancer patients similarly found that a substantial proportion of informal caregivers face substantial subjective burden and are simultaneously faced with decreased quality of life, increased risk of depression, and a decreased ability to work or engage in normal activity [56–58].

Our results showed that younger informal caregivers (65 and below years) had significantly higher ZBI scores than their older counterparts, which is in line with Cain and Wicks’s study [59, 60]. Younger informal caregivers must fulfill varied and at times conflicting responsibilities in caregiving, work, and social life. We also found that spouse caregivers experience a significantly higher subjective burden than other relatives who provide care, which is consistent with studies of India and rural China though contrary to a Dutch study [34, 43, 46]. This finding was not surprising in a Chinese context, since spouses who live with their patients would face fewer role conflicts. However, other family members who care for patients with critical illnesses may experience subjective burdens due to their variable family roles [46, 61]. Parental caregivers experience more anxiety, and child caregivers face varied pressures such as weakened social support, lower income, and conflicting demands on time from work and childcare.

Our results displayed the same codirectional relationships between objective and subjective burdens associated with background factors found in previous studies [33, 62, 63]. We found that 54.69% of caregivers cared for patients for more than 8 hours per day, and 36.64% cared for patients for more than 12 hours per day. This substantial time investment limits self-directed activities such as socialization, entertainment, and other activities, let alone normal work [20, 64]. Prolonged periods of mental stress, physical exhaustion, and lack of social support can impose a financial and psychological burden on caregivers, often lasting months or years [65]. Though long-term care insurance is increasingly available for urban employees, most rural patients in China often lack coverage for their post-discharge care and minimal support for family caregivers. Caregivers receive minimal support of any kind, either in their capacity as caregiver or as an individual [66].The situation is even worse for caregivers who are themselves sick [67]. That said, providing home inpatient support for family caregivers has been shown to substantially relieve psychological stress and to improve caregivers’ health and quality of life [11]. Additionally, a study of 18 European countries found that availability of formal long-term care resources mitigated the negative externalities of informal care [68]. In light of these findings, we recommend exploring more caregiving channels, expanding the types of services and the duration of care provided by health care systems, and increasing the psychological care and skill training available to caregivers [11, 69, 70]. Such an approach could mitigate stress and perceived burdens for caregivers and improve patient and family outcomes [43, 71].

Our study found that financial burdens worsen subjective burdens. Higher OOP costs and lower income levels generate higher burdens, as noted in previous studies [72]. Unexpectedly, however, caregivers for families with debt had lower subjective burdens. This may be explained by the fact that families with debt have better social relationships relative to other families, possibly enjoying financial support from relatives and friends [73]. Moreover, low incomes and poverty may generate pessimism, frustration, and interpersonal anxiety and rejection, attitudes which may prevent effective engagement with potential social support mechanisms [26, 74]. Support from relatives, friends, communities, and village doctors may mitigate these negative attitudes [75]. Furthermore, increased socioeconomic support or insurance for informal family caregivers could improve patient and family outcomes [76].

Medical aid systems can also reduce caregiving burdens [77, 78]. A Nepalese study found that community-based health insurance and accessible medicine reduce the economic burden associated with disease [79]. Unfortunately, healthcare coverage in rural Shandong is limited, and only 6.32% of our sample patient population had access to such coverage. In China, critical illness insurance for diseases with high OOP costs began in 2012 for rural residents, and was expanded to cover all urban and rural residents in 2015 [80, 81]. However, our study found no correlation between critical illness insurance policies and subjective caregiver burdens, possibly because critical illness insurance does not cover post-discharge medical care and associated costs. Our study suggests that insurance should cover both direct medical burdens and attempt to mitigate the indirect economic burdens and intangible psychological burden of major diseases. Third-party liability insurance may reduce the economic burden of critical illnesses [79].

### Limitations

Because of the cross-sectional design, causality cannot be inferred in this study’s results, since only correlates can be examined. In addition, because the study encompassed a larger number of diseases and had limited samples in each category, there is no classification analysis to analyze the caregiver burden in different situations. Because the subjects of our study were rural residents, the results may be affected by self-reported measurement bias and may not be easily generalized. Despite these limitations, our study clearly illustrates the subjective burdens of caregivers of critically ill patients in rural areas. Future research may further explore the role of each factor.

## Conclusions

Informal caregivers of patients with critical illnesses are confronted with heavier subjective burdens than other caregivers, due to stress produced by lengthy care, emotional exhaustion, and financial burdens shared with the patients. Optimal insurance and social policies should address both direct medical burdens as well as the intangible psychological burden of critical diseases. We therefore recommend (1) building a social support system for patients with critical illness beyond simple financial support, (2) focusing on caregivers' mental health and conducting early interventions on their behalf, and (3) expanding long-term care insurance to facilitate formal care.

## Data Availability

The data used during this study are available from the corresponding authors on reasonable request.

## Abbreviations

ZBI: Zarit Burden Interview
OOP: Out-of-Pocket
